# Case Report: Primary Squamous Cell Carcinoma of the Orbit in a Patient With Carney's Syndrome Treated With Multidisciplinary Approaches

**DOI:** 10.1002/cnr2.70020

**Published:** 2024-09-26

**Authors:** Md. Arifur Rahman, Rajesh Balakrishnan, Mohammad Golam Mostofa, Mohammed Rashedul Islam, Enamul Kabir, Md. Shariful Islam, Bidoura Naznin, Arunangshu Das, Qamruzzaman Chowdhury

**Affiliations:** ^1^ Department of Oncology Bangladesh Specialized Hospital Limited Dhaka Bangladesh; ^2^ Department of Radiation Oncology CMC Vellore Vellore India; ^3^ Department of Histopathology Anowara Medical Service Dhaka Bangladesh; ^4^ Department of Plastic Surgery BIRDEM General Hospital Dhaka Bangladesh; ^5^ Department of Physics Delta Medical College Hospital Dhaka Bangladesh; ^6^ Department of Oncology Square Hospitals Limited Dhaka Bangladesh

**Keywords:** cardiac myxoma, Carney's syndrome, papillary carcinoma of thyroid, primary orbit cancer, squamous cell carcinoma

## Abstract

**Background:**

Squamous cell carcinoma (SCC) is a rare malignancy of invasive epithelium with keratinocyte differentiation, and it is the most common form of eyelid malignant neoplasm, comprising 5%–10% of malignancies. While SCC rarely affects the orbit, it may be involved through local invasion from a cutaneous primary site or extension by perineural invasion. Only 12 cases of primary orbital SCC have been reported until now. Here, we present a case of primary carcinoma of the right orbit with coexisting Carney's syndrome, a rare genetic disorder associated with multiple endocrine neoplasias (MEN) syndromes.

**Case:**

A 62‐year‐old South Asian male presented with a painful swelling in the lateral aspect of the right eyebrow and protrusion of the eyeball in August 2020. He had a history of excision of Right atrial Myxoma in March 2020. Orbital computerized tomography (CT) and positron emission tomography (PET‐CT) scans revealed an enhancing soft tissue lesion in the right orbit with the involvement of frontal and ethmoid sinuses. Biopsy confirmed HPV‐related poorly differentiated SCC, positive for HPV‐related markers. The patient received concurrent chemo irradiation with Cisplatin. Follow‐up PET‐CT done 3 months later showed a new lesion appeared in the right orbital region and right lobe of thyroid. Later had surgical excision and total thyroidectomy, and histopathological examination (HPE) from orbit was reported as invasive SCC and from the thyroid was reported as synchronous papillary thyroid cancer. The patient's proptosis resolved, and subsequent PET‐CT and magnetic resonance imaging (MRI) scans did not show any residual or recurrent disease.

**Conclusion:**

Primary SCC of the orbit is an extremely rare disease, and this case report presents the 13th reported case and the first one associated with Carney's syndrome. As there is no standard treatment regimen for primary SCC of the orbit, this case highlights the use of multimodality treatment, including surgical excision and chemo irradiation. The findings emphasize the importance of early detection and management of this uncommon and life‐threatening condition, providing hope for patients and aiding in the prevention of recurrence.

## 1 Introduction

Primary orbital squamous cell carcinoma (SCC) is an exceedingly rare condition due to the absence of native squamous epithelium within the orbit. SCC, the most common form of malignant eyelid neoplasm, accounts for 5%–10% of such malignancies and typically affects individuals aged 50–75. Despite this, the incidence rate of orbital SCC is very low, ranging from 0.09 to 2.42 cases per 0.1 million population [1, 2, 3]. The few reported cases pose significant diagnostic challenges due to their nonspecific symptoms. Furthermore, treatment is complex, particularly when using irradiation and organ preservation modalities, requiring precise dose constraints for vital structures such as the opposite eye, lens, retina, optic nerve, and optic chiasm.

This case is unique due to its association with Carney's syndrome, a rare genetic disorder that adds further complexity to the clinical picture. The co‐occurrence of primary orbital SCC and Carney's syndrome in a single patient is highly unusual. We present this case to detail the diagnostic workup, staging, and multidisciplinary management approach, emphasizing the need for a comprehensive and collaborative strategy in treating such rare and complex malignancies.

## 2 Case Presentation

**FIGURE 1:**
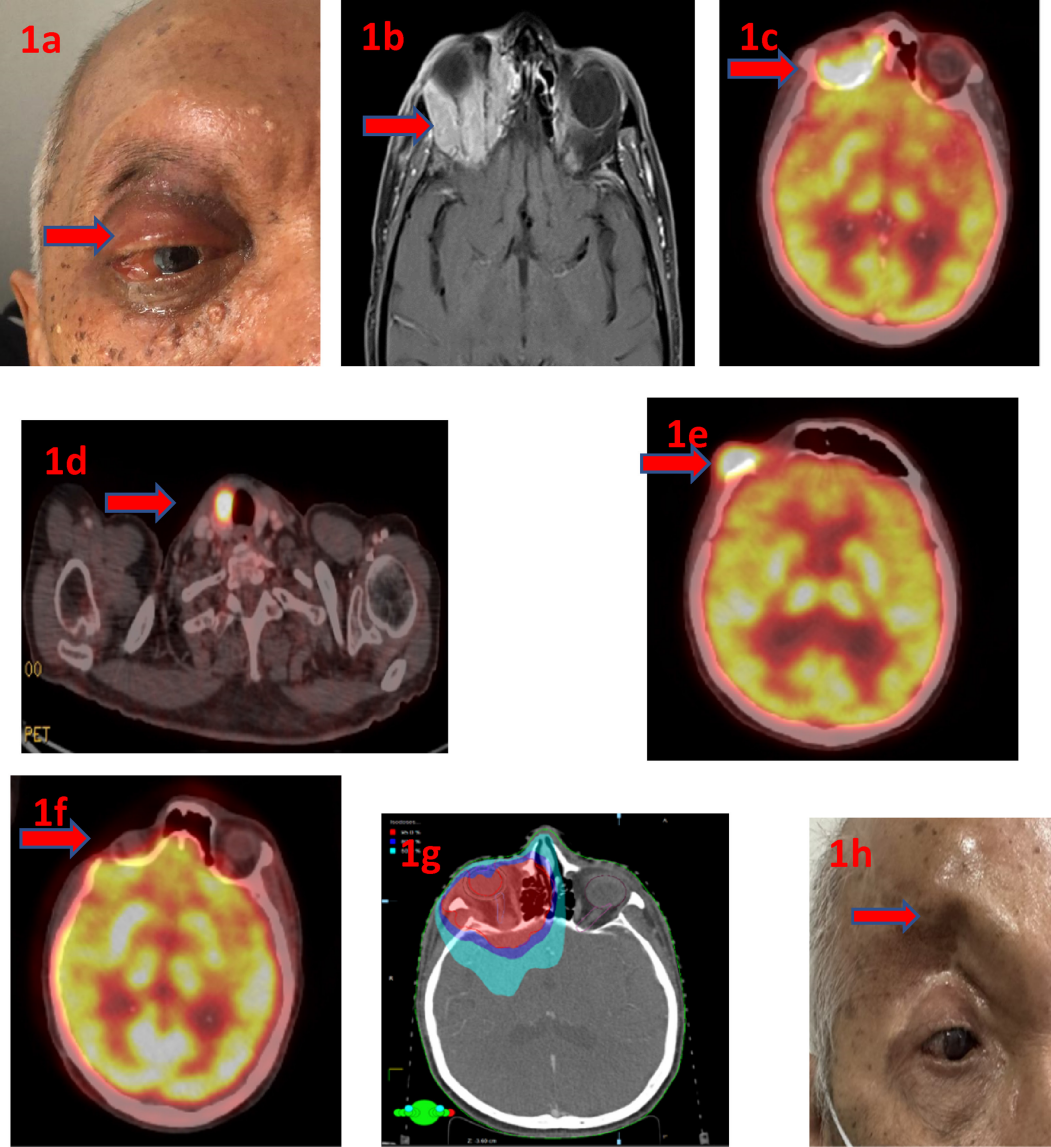
(a) Patient photograph during treatment, showing edematous conjunctiva and swelling. (b) Arrow marked showing hyperintense (T1 contrast of MRI orbit after post‐excision biopsy) soft tissue mass involved the retro‐orbital right side area with extension to medial and lateral aspects. (c and d) Arrow marked region showing the FDG avid (PET‐CT images before treatment) area in the right side of the orbit (c) with FDG avid region in the right lobe of thyroid (d). (e) After CCRT and before reconstructive surgery, arrow mark area showing high FDG avid region of residual disease. (f) Arrow marked area showing no regional FDG uptake (follow‐up PET scan after 2.5 years of treatment) in the primary right orbit. (g) Rapid arc dose color wash during radiotherapy showing coverage in PTV, 95% in red, 80% in blue, and 50% in turquoise color. (h) Recent photograph during last follow‐up visit in July 2023, 3.3 years after treatment.

**FIGURE 2:**
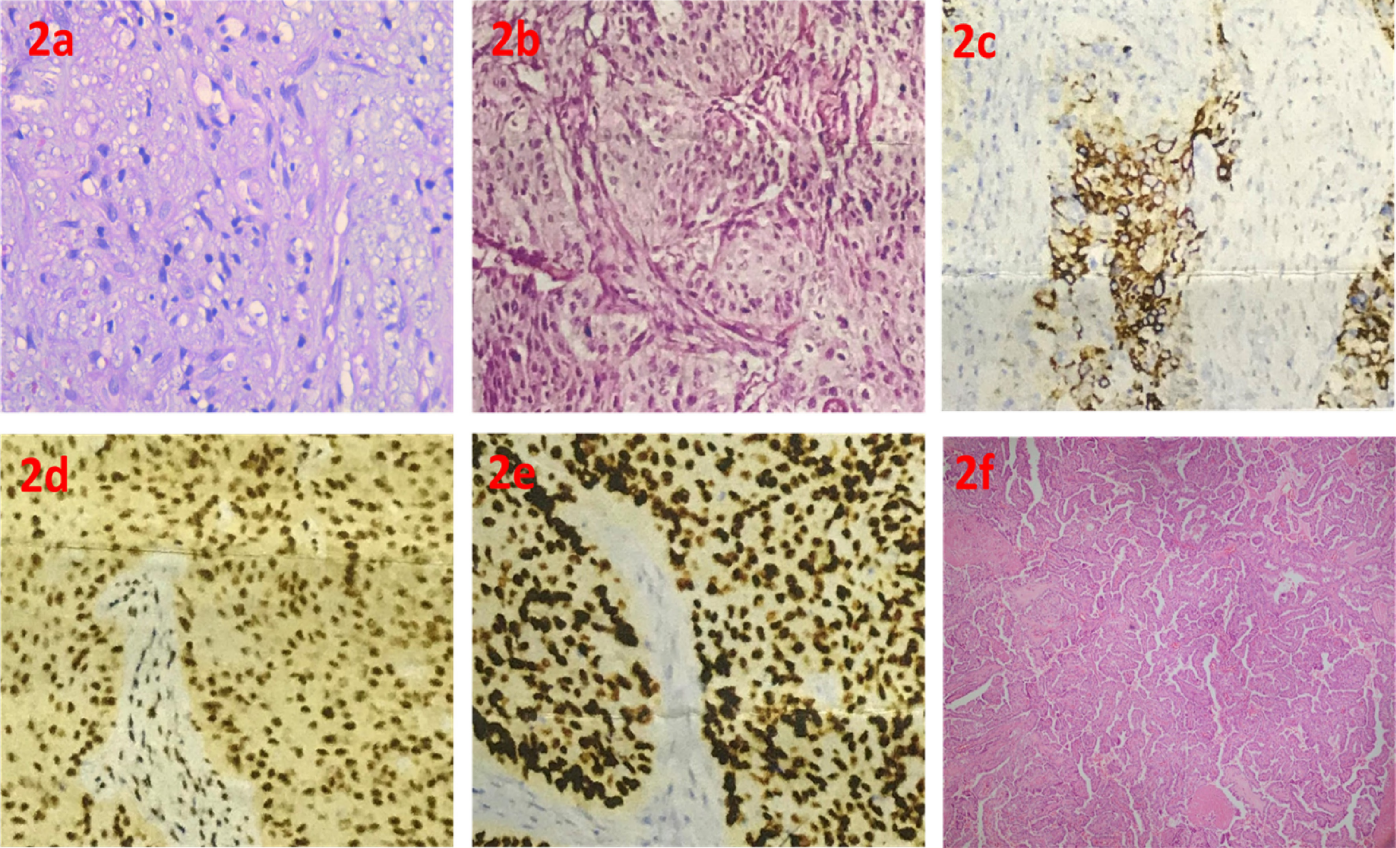
(a) Myxoma, H&E stain, high power image (40×) showing spindle‐shaped cells in a myxoid background. (b) Orbital tissue, H&E stain, high power image (40×) showing squamous cells carcinoma, Grade II. (c) Orbital tissue, IHC of CK5/6 in high power image (40×) showing tumor cells is positive. (d) Orbital tissue, IHC of p16 in high power image (40×) showing tumor cells is positive. (e) Orbital tissue, IHC of p63 in high power image (40×) showing tumor cells is positive. (f) Total thyroid specimen (right lobe) H&E stain, low power image (10×) showing papillary carcinoma thyroid, classic.

A 62‐year‐old South Asian male (Figure 1a) presented in August 2020 with painful swelling in the lateral aspect of the right eyebrow and protrusion of the eyeball. His medical history included the excision of a right atrial myxoma in March 2020 (Figure 2a) performed at United Hospitals Limited, Dhaka, Bangladesh. Clinical examination revealed proptosis, a tender mass fixed to the skin. Orbital computed tomography (CT) indicated an enhancing soft tissue lesion measuring 3.2 × 3 cm^2^ in the superior‐medial aspect of the right orbit, with erosion of the orbital roof and medial nasal bone, and extension into the frontal and anterior ethmoid sinuses. The lesion was extraconal, abutting the superior rectus, compressing the right globe, and sparing the lacrimal gland. Staging positron emission tomography (PET)‐CT identified a hypermetabolic soft tissue mass in the superior‐medial aspect of the right orbit (Standard Uptake Value [SUV] max 19.95) involving the right frontal sinus, eroding the orbital roof, and extending to the ethmoid sinus, suggestive of malignancy (Figure 1c). Additionally, a hypermetabolic nodule was found in the right lobe of the thyroid (SUV max 13.4) (Figure 1d), with no evidence of distal metastases in the lungs, liver, bone, or lymph nodes.

An incisional biopsy of the right orbital mass was performed at Bangladesh Eye Hospitals, with histopathology revealing poorly differentiated SCC (Figure 2a). A second opinion confirmed this diagnosis, with immunohistochemistry showing CK5/6‐positive (Figure 2c), p16‐positive (Figure 2d), and p63‐positive (Figure 2e) markers, indicating HPV‐related SCC. Fine‐needle aspiration cytology (FNAC) of the thyroid nodule suggested papillary thyroid carcinoma. A postoperative magnetic resonance imaging (MRI) in September 2020 (Figure 1b) displayed a large heterogeneous mass (3.6 × 4.6 × 3.3 cm^3^) in the superior‐medial region of the right orbit, extending into the right frontal and ethmoid sinuses, involving the superior rectus muscle but not reaching the apex, causing significant proptosis.

The case was discussed in a multidisciplinary team (MDT) meeting at Bangladesh Specialized Hospital Limited, given the rare diagnosis. It was decided to pursue concurrent chemoradiation (CCRT) with Cisplatin for organ preservation. Radiotherapy was administered at Delta Medical College Hospital, Dhaka, Bangladesh, using the RapidArc technique with a Varian TrueBeam machine (Version 2.7), delivering a dose of 56 Gy in 28 fractions (200 cGy per fraction) along with six doses of concurrent Cisplatin (30 mg/m^2^) on a weekly schedule. Adaptive re‐planning was necessary due to shrinkage of the orbital swelling during treatment. Concurrent CCRT was completed in October 2020, with the dose‐volume histogram (DVH) and maximum dose (cGy) parameters for the planning target volume (PTV‐56) and organs at risk (OAR) as follows: PTV‐56: 6049.1, brainstem: 2174.7, left cochlea: 141.4, right cochlea: 639.5, left eye: 889.1, right eye: 5983.7, left lens: 252.1, right lens: 5346.1, optic chiasm: 3688.1, right optic nerve: 5976.2, left optic nerve: 2203.5, and pituitary: 2992.4.

Three months post‐CCRT, the patient developed an exophytic growth in the lateral aspect of the right orbit. A repeat PET‐CT (Figure 1e) showed a 19 × 13 mm^2^ hypermetabolic lesion (SUV max 11.25) in the right anterior lateral angle of the orbit. The frontal sinus, superior‐medial aspect of the right orbit, and ethmoid sinus showed no uptake, confirming a good response to CCRT. However, the hypermetabolic lesion in the right thyroid lobe persisted (SUV max 13.12). The MDT re‐evaluated the case, and the patient underwent excision of the new orbital lesion with reconstruction, right eye tarsorrhaphy, and total thyroidectomy in January 2021 at Square Hospitals Limited. Histopathological examination revealed invasive SCC of the right orbit with deep margin involvement, bone involvement, and uninvolved frontal sinus tissue. The total thyroidectomy specimen confirmed papillary carcinoma of the right thyroid lobe (Figure 2f) (2 cm, clear margins, 1 lymph node with tumor, lymphovascular space invasion present—pT1N1aMx).

At the last clinical follow‐up in January 2024, the patient had near‐normal vision in the left eye and poor vision in the right (finger counting). Proptosis had resolved, and consecutive PET‐CT (Figure 1f) and MRI scans, the latest in December 2023, showed no residual or recurrent disease (Figure 1h).

## 3 Discussion

SCC of the orbit is an extremely rare disease, with only 12 cases reported to date. Our case represents the 13th documented instance and the first case of a patient with Carney's syndrome (Table 1). Despite its rarity, orbital SCC is progressive and life‐threatening. Our patient, Mr. Kaiser, a 62‐year‐old male, presented with carcinoma of the right orbit, which included bone erosion and involvement of the frontal and ethmoidal sinuses. He initially visited with symptoms of a burning sensation, itching, and watering from both eyes. Examination revealed painful swelling on the lateral side of the right eyebrow and protrusion of the right eyeball.

**TABLE 1:** Literature data collection and case study.

Citation	Author/year	Age/sex	Side	Site of lesion	Surgery	RT	Chemo	CCRT	Response to treatment
[4]	Ruff et al. 1985	53/F	Left	Inferotemporal orbit	Yes	Yes	No	No	CR at 84 months
[5]	Saha et al. 2011	56/F	Right	Orbital apex	No	Yes	No	No	CR at 72 months
[6]	Peckinpaugh et al. 2012	43/F	Right	Orbital apex	No	Yes	Yes	No	CR at 48 months
		63/M	Left						Died after 19 month
		67/M	Right						Died after 12 months
[7]	Hromas and Sokol 2014	63/M	Left	Inferior interconal space	Yes	Yes	No	No	CR at 12 months
[8]	Choi et al. 2014	74/F	Left	Superomedial orbit	Yes	Yes	No	No	CR at 17 months
[9]	Arbulu et al. 2017	73/F	Right	Superior orbit	Yes	Yes	No	No	CR at 34 months
[10]	Blandford et al. 2018	63/M	Right	Superomedial orbit	Yes	Yes	Yes	No	CR at 12 months
[11]	El Samkary et al. 2021	99/F	Left	Superolateral orbit	Yes	No	No	No	CR at 6 months
[12]	Karrabi et al. 2022	45/F	Right	Both extraconal and Intraconal mass	Yes	Yes	Yes	Yes	Not reported
[13]	Krishnamurthy et al. 2023	56/F	Left	Both intraconal and extraconal	No	Yes	No	No	CR at 24 months
New	Rahman et al. (Our case)^a^ 2024	62/M	Right	Superolateral orbit	Yes	Yes	No	Yes	CR at 36 months

^a^ Ours is the first case of orbital tumor in a patient with Carney's syndrome.

A 2012 case report by Peckinpaugh et al. described a 63‐year‐old male patient presenting with diplopia, ophthalmoplegia, eye pain, and forehead numbness [6]. In our case, the soft tissue mass measured 3.2 cm in the superior‐medial aspect of the right orbit. The lesion was extraconal, abutting the superior rectus muscle and compressing the right globe, which abutted the sclera. A similar case reported in 2017 by Campos Arbulu et al. involved a 73‐year‐old patient with a 3.5‐cm lesion located in the superior orbit, abutting the frontal sinus [14]. Another 2012 report by Peckinpaugh et al. described a 43‐year‐old female patient with a lesion located in the orbital apex, though the size was not reported [6].

Diagnosis in our case was based on clinical examination and various imaging modalities, including CT‐Scan, PET CT‐Scan, and excision biopsy. A 2011 case report by Saha et al. detected the disease using an MRI of the brain, orbit, and sinus (initially negative), repeated MRI 1 year later, excisional biopsy via transfrontal craniotomy, and systemic evaluation by a clinical oncologist [5].

The management of orbital SCC is challenging due to issues of inoperability or incomplete excision. In our case, bone erosion was present, and although SCC typically requires a radiotherapy dose escalation up to 60–66 Gy [15], we were only able to deliver 56 Gy. The appropriate guidelines for concurrent chemotherapy are not established, and there is no evidence of neoadjuvant treatment reported. Treatment for the coexistent papillary thyroid cancer and follow‐up is also complex. Our patient underwent excision of the right atrial myxoma, excision biopsy, concurrent chemoradiotherapy, plastic surgical reconstruction due to recurrence, and total thyroidectomy for synchronous papillary thyroid cancer. A 2018 case report by Blandford et al. described a 63‐year‐old male patient treated with right orbitotomy with excisional biopsy and orbital chemo irradiation [10]. In our case, recurrence surgery was challenging due to local failure and the need for organ preservation, necessitating the involvement of plastic and reconstructive surgeons, as well as ENT and head and neck surgeons. Post‐excision biopsy showed bone invasion and deep margin involvement, making further adjuvant treatment challenging despite the ypT4 disease with high Grade 3. Options such as short‐duration radiotherapy intervals and re‐radiation with conventional proton therapy were considered, but the MDT decided on observation rather than further adjuvant treatment.

Carney's complex is a rare genetic disorder associated with MEN syndromes, affecting multiple glands in the body, such as the thyroid, pituitary, and adrenal glands, and causing cardiac myxomas [16]. An important aspect of this case is that the patient had atrial myxoma, orbital SCC, and papillary carcinoma of the thyroid almost simultaneously. He fulfilled two of the major criteria required for the diagnosis of Carney's complex [17]. Carney's complex predisposes individuals to develop multiple neoplasms, necessitating regular follow‐up and early screening for new tumors.

As far as we know, this case is the first to report the development of orbital SCC in a patient with Carney's complex. Recognizing Carney's complex is crucial as it predisposes patients to multiple future neoplasms, necessitating regular follow‐up and early tumor screening. Identifying mutations in the protein kinase A Type I alpha regulatory subunit (PRKAR1A) is essential for the patient and potentially affected at‐risk family members [18]. Our patient has not been tested for these mutations due to the lack of facilities in our country. Malignant large‐cell calcifying Sertoli cell tumors, pituitary adenomas, and melanotic schwannomas are commonly associated with Carney's complex and should be considered during follow‐up.

## 4 Conclusion

There is no established standard treatment protocol for primary SCC of the orbit, given its rarity and varied clinical presentations. From primary orbital radiation and observation to orbital exenteration with adjuvant chemotherapy and radiation, various approaches have been explored in the literature. In our case, we utilized a multimodal treatment approach, including surgical excision, chemotherapy, and radiotherapy, which contributed to improving the patient's survival.

This case underscores the importance of early diagnosis and a multidisciplinary treatment approach in managing primary orbital SCC, especially in the context of concurrent Carney's syndrome. Our findings highlight the need for further research and awareness to improve understanding of this rare disease and develop effective treatment strategies. By sharing our experience, we aim to raise awareness about this rare and aggressive malignancy, offer hope to patients facing a challenging diagnosis, and contribute to efforts in preventing recurrence and improving patient outcomes in the future.

## Author Contributions

**Md. Arifur Rahman:** conceptualization, investigation, writing – review and editing, writing – original draft, supervision, methodology, validation, data curation, resources, visualization. **Rajesh Balakrishnan:** investigation, writing – review and editing, visualization, formal analysis, supervision, methodology, validation, data curation. **Mohammad Golam Mostofa:** supervision, writing – review and editing, investigation. **Mohammed Rashedul Islam:** investigation, writing – review and editing, visualization. **Enamul Kabir:** investigation, visualization, writing – review and editing, methodology. **Md. Shariful Islam:** investigation, writing – review and editing, formal analysis. **Bidoura Naznin:** investigation, writing – review and editing, formal analysis, supervision, resources. **Arunangshu Das:** investigation, writing – review and editing, visualization. **Qamruzzaman Chowdhury:** writing – review and editing, methodology, supervision, investigation.

## Ethics Statement

Written informed consent was obtained from the patient for the publication of case details and the use of images.

## Conflicts of Interest

The authors declare no conflicts of interest.

## Data Availability

The data that support the findings of this study are available on request from the corresponding author. The data are not publicly available due to privacy or ethical restrictions.
